# A Cheerful Home

**Published:** 1884-09

**Authors:** 


### A CHEERFUL HOME.

THE BITTERNESS of a single unkind word may disquiet an entire family for a whole day. One surly glance casts a gloom over the household, while a smile, like a gleam of sunshine, may light up even the darkest and weariest hours. Like unexpected flowers which spring up along our path, full of freshness, fragrance and beauty, do kind words and gentle acts and sweet dispositions, make glad the home where peace and blessings dwell. No matter how humble the home, if it be thus garnished with grace and sweetened with kindness and smiles, the heart will turn lovingly toward it from all the tumult of the world; it will be the dearest spot beneath the circuit of the sun. And the influences of home perpetuate themselves.

The gentle grace of the mother lives in the daughter long after her head is pillowed in the dust of earth; and the fatherly kindness finds its echo in the nobility and courtesy of sons who come to wear his mantle and fill his place; while, on the other hand, from an unhappy, misgoverned and disordered home go forth persons who shall make other homes miserable, and perpetuate the sourness and sadness, the contentions and strifes and railings which have made their own early lives so wretched and distorted.

Toward the cheerful home the children gather "as clouds, and as doves to their windows," while from the home which is the abode of discontent and strife and trouble, they fly forth as vultures to rend their prey.

The class of men who disturb and distress and disorder the world, are not those born and nurtured among the hallowed influences of happy homes; but rather those whose early lives have been a scene of trouble and vexation—who have started wrong in the pilgrimage, and whose course is one of disaster to themselves, and trouble to those around them.

